# Blue rubber bleb nevus syndrome in a Malay girl: A case report and literature review

**DOI:** 10.1016/j.ijscr.2020.05.036

**Published:** 2020-06-01

**Authors:** Fatimah Mat Johar, Wan Azman Wan Sulaiman, Arman Zaharil Mat Saad, Normala Basiron, Nik Amin Sahid

**Affiliations:** a Reconstructive Sciences Unit, School of Medical Science, Universiti Sains Malaysia, 16150 Kubang Kerian, Kelantan, Malaysia; b Hospital Universiti Sains Malaysia, Jalan Raja Perempuan Zainab II, 16150 Kubang Kerian, Kelantan, Malaysia; c Plastic and Reconstructive Surgery, Hospital Kuala Lumpur, 23, Jalan Pahang, 50586 Kuala Lumpur, Wilayah Persekutuan Kuala Lumpur, Malaysia; d Department of Surgery, Faculty of Medicine and Health Sciences, Universiti Malaysia Sabah, Sabah, Malaysia

**Keywords:** Blue rubber bleb nevus syndrome, Venous malformation, Anomalies, Sclerotherapy

## Abstract

**Introduction:**

Blue Rubber Bleb Nevus Syndrome (BRBNS) also known as Bean’s Syndrome is an atypical type of vascular malformation. To date, around 200 cases have been reported world-wide. In view of its low incidence rate, clinicians might misdiagnose and under treat. The key features of this syndrome are characterized by multiple cutaneous, soft tissue and gastrointestinal tract venous malformations.

**Presentation of case:**

We report the first case of Blue Rubber Bleb Nevus Syndrome in Malaysia, a 23 years old Malay girl who suffers from multiple cutaneous venous malformation and gastrointestinal bleeding episodes.

**Discussion:**

The typical morbidity for this syndrome is symptomatic anemia due to secondary iron deficiency due to the gastrointestinal venous malformation bleeding. In managing the gastrointestinal bleeding, it mainly depends on the severity of gastrointestinal bleeding, some may resolve spontaneously, while the others may be needing blood transfusion, and some may require GIT resections. As for cutaneous lesions, normally it is innocuous depending on the region and size. Large or problematic cutaneous venous malformation might benefit from sclerotherapy or excision.

**Conclusion:**

Multidisciplinary approach is crucial in managing BRBNS case due to its complexity and the spectrum of multiple organ involvement to ensure the best outcome to the patient.

## Highlights

• BRBNS also known as Bean’s Syndrome are atypical type of vascular malformation which uncommon.
• The key features of this syndrome is characterized by multiple cutaneous, soft tissue and gastrointestinal tract venous malformation.
• Current treatment modality for this rare disease is based on the severity of the organ involvement. It may involve minimaly invasive technique or extensive surgical resection.

## 1 Introduction

Vascular anomalies as classified by Mulliken and Glowacki in 1982, are divided into 2 groups which are the hemangiomas and vascular malformations based on their cellularity features [1]. Over the years, the classification underwent refinement which currently divided into vascular tumors and vascular malformations as proposed by the International Society for Vascular Anomalies in 1996 [2]. Current classification has made diagnosis, treatment and prognosis more structural.

Blue rubber bleb nevus syndrome (BRBNS) was first found by English dermatologist George Gaskoin in 1818 [3]. As 100 years passed, Bean coined the term Blue rubber bleb nevus syndrome as he was the first person to describe the abnormality. Up to this date, only around 200 published case reports regarding this abnormality [4]. From the data that was published by X.L Lim et al., they found 20% of patients with BRBNS were from the United States, 15% from Japan, 9% from Spain, 9% from Germany, 6% from China, and 6% from France. There are also reports from other countries; however, the number of cases is much lower [4]. However up to date, there is no case reported from Malaysia regarding BRBNS.

Blue rubber bleb nevus syndrome is depicted by cutaneous and internal visceral venous malformation. Most common internal visceral involvement is the gastrointestinal organs. Morbidities that may arise are gastrointestinal hemorrhage, intussusception, volvulus, internal hemorrhage and infarction. Other organ involvement such as the central nervous system, liver, kidney, bladder, heart, thyroid, and spleen has been reported as well [5].

This disease is sporadic however there are reports regarding genetically link via autosomal dominant and linkage to a locus on chromosome 9p [6]. We report a case of a 23 years old Malay lady with multiple cutaneous venous malformation and gastrointestinal bleeding. This is the first Malay patient with BRBNS reported in English literature and her case has been reported in line with the SCARE criteria [7].

## 2 Case report

A 23 years old Malay lady who was born with a solitary lesion over her right foot dorsum. Initially the size of the lesion was about the size of a pea and gradually increase in size and causing pain. At the age of 1 month old, parents claimed it was excised. Over the years, other cutaneous skin lesion erupted and size gradually increase. The biggest lesion was over the right arm and it was operated at the age of 2 in tertiary hospital by hand surgeon.

Patient had her episodes of recurrent intestinal bleeding which resolves spontaneously since the age of 19 and requires frequent blood transfusions. Later, the patient was referred to surgeon for her chronic anemia with multiple cutaneous bluish lesion. Investigations that was done including OGDS, colonoscopy, capsule endoscopy and CT abdomen. Due to this indwelling problem, patient underwent laser treatment of the gastrointestinal lesion and she was symptom free for about one year. Sclerotherapy for lesions on each foot sole and right hand dorsum had to be done due to pain especially upon ambulation.

Unfortunately a year later, patient had a massive gastrointestinal bleeding which require patient to underwent major surgery. Intraoperative findings were multiple naevi over small and large bowel with contact bleeding which were excised, large naevus at the mesentery at the proximal jejunum which was left in-situ, naevus over the appendix and appendicectomy was done and multiple naevi over the liver surface which was left in-situ. A total of 53 enterotomies and 9 colotomies was done for this patient. On the same setting of the surgery, over 112 cutaneous lesions were excised over suprapubic region and bilateral thighs. Post operatively she was recovered from any intestinal bleeding and does not require any blood transfusion. Clinically she was well and pink, laboratory investigations shows her hemoglobin level maintain at 10 gm/dL.

A year later, patient complaining of bilateral cheek and intraoral lesions which has cause occasional pain and gum bleeding. Case was evaluated by plastic surgeon and multiple cutaneous lesions were noted over bilateral cheek, over the gums, lingual and sublingual, bilateral palms and soles, over the abdomen and back. The lesions were bluish to grey in color, soft and compressible in nature. MRI of head and neck as well as MRA of brain and cervical was done for her. Findings of the imaging were numerous venous malformation over pterygoid muscle, masseter muscle and infratemporal fossa, nasopharyngeal mucosal space, parapharyngeal space, bilateral parotid, submandibular space, sublingual space, soft tissue of the cheek, supraclavicular fossa, thyroid gland, scalp, superior and middle mediastinum. Intraoral lesions are over soft palate, intrinsic and extrinsic tongue muscles till floor of the mouth (Fig. 5).

**Fig. 1:**
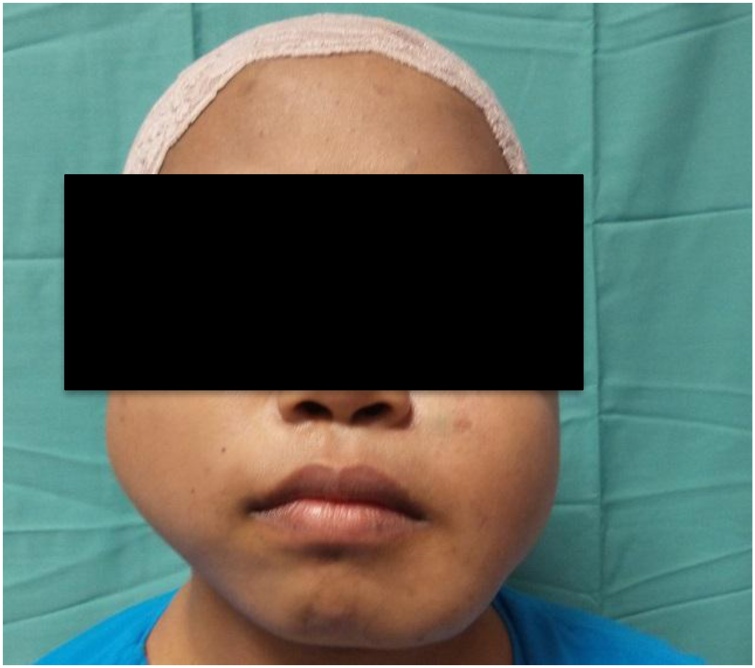
Photo above is showing 3 month post first sclerotherapy and bilateral cheek lesions clinically still detectable.

**Fig. 2:**
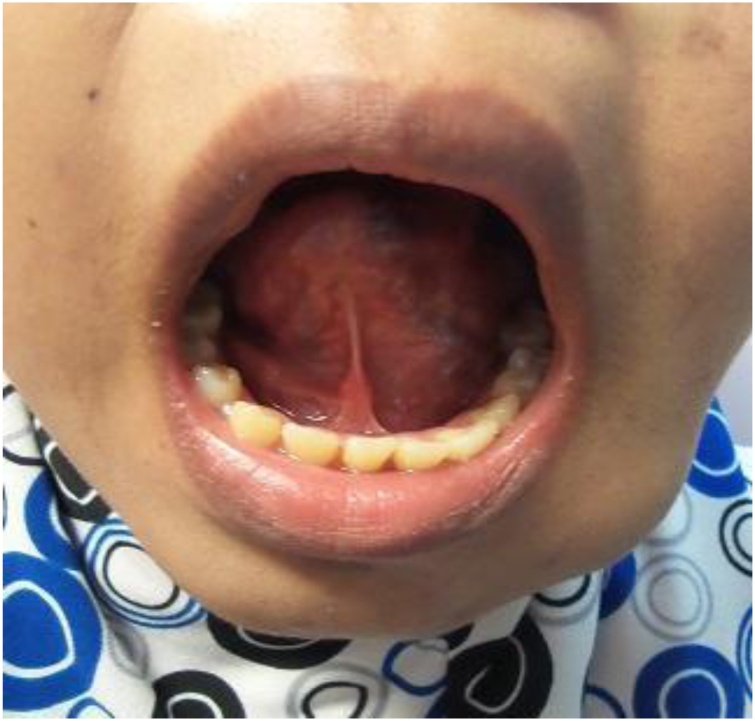
Photo above is showing multiple bluish sublingual lesions.

**Fig. 3:**
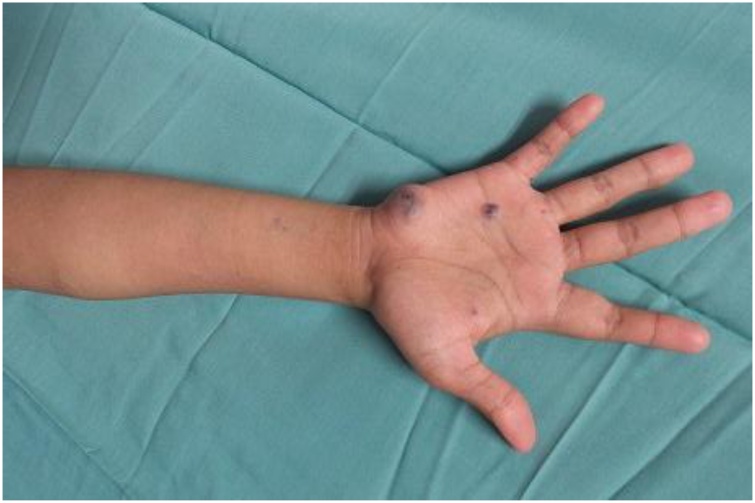
Photo above is showing multiple bluish cutaneous lesions over right palm and forearm.

**Fig. 4:**
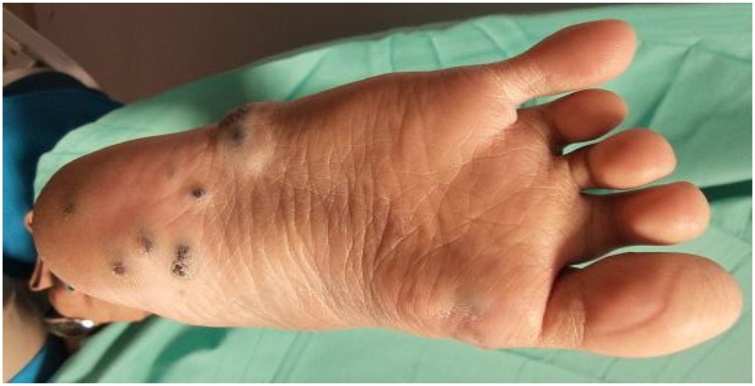
Photo above is showing multiple bluish cutaneous lesion of right foot sole.

At the age of 23, patient underwent sclerotherapy of bilateral cheek lesions. Sodium tetradecyl and Lipiodol was injected into both lesions by the help of our Interventionist Radiology. Post sclerotherapy two month later, size of the bilateral cheek lesions has reduced however the lesions was still present (Fig. 1, Fig. 2, Fig. 3, Fig. 4). Patient underwent another session of sclerotherapy 3 month later and the lesions has shrunk further more. Patient is happy with the outcome and still under plastic surgery team follow up.

**Fig. 5:**
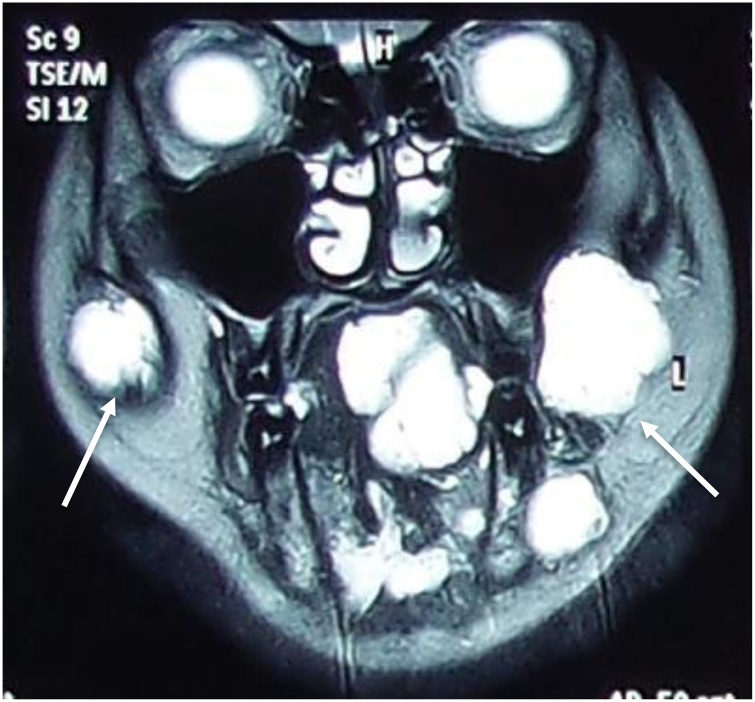
MRI above is showing multiple lobulated lesions over bilateral cheek soft tissue and tongue (white arrow).

## 3 Discussion

Vascular malformation is due to defects in vascular development during vasculogenesis and especially during angiogenesis phase [8]. Vasculogenesis is a process of vessel growth from embryonic cells which is the mesoderm which later will differentiate as angioblasts (endothelial precursors) and hemocytoblasts (blood cell precursors) [8]. Angioblast fusion occurs in the vascular islets, forming the primary capillary plexus. This primary capillary system will extend and mature during angiogenesis, which involves both mural cell recruitment and endothelial cell proliferation to generate the fully developed and functional lymphatic and vascular trees. Angiogenic factors, such as angiopoietins (ANGPT-1 and ANGPT-2), fibroblast growth factors, vascular endothelial growth factors (VEGFs) and platelet derived growth factor beta (PDGF-beta) regulate angiogenesis. These factors activate precursor cells, and stimulate migration, proliferation, and differentiation of the primary capillary plexus [8].

Venous malformations are classified into sporadic venous malformations (VM), dominantly Cutaneo-mucosal venous malformations (VMCM), and dominantly inherited glomuvenous venous malfromation (GVM) [8]. VMCMs are characterized clinically by multifocal, hemispherical and small bluish lesions (<5 cm in diameter). Tissue planes involvements are the skin and oral mucosa, also may invade superficial muscle, lungs, gastrointestinal tract and brain [8]. The features mentioned about VMCM are similar to BRBNS, therefore we conclude that they are no different from one and another.

BRBNS has three clinical types (Table 1). The most common type is type II, clinically it appears as thin-walled, bluish, blood-filled sac, as shown in this case report [9]. The lesion is compressible and refills slowly once pressure is released. However our patient appear to have overlapping clinical type. The lesions may associate with pain and hyperhidrosis. The excessive sweating is due to close association with the sweat glands in the skin overlying [5,9].

**Table 1:** Classification of Blue Rubber Bleb Nevus Syndrome.

Types	Clinical Findings
I	Large disfiguring venous malformation that obstructs vital tissues
II	'Blue rubber nipples' – bluish, thin-walled, blood-filled sac. This lesion is easily compressed and refills slowly upon release of pressure, and it usually presents with associated pain and hyperhidrosis
III	Irregular blue-black macule or plaque that may be punctated and rarely blanches on pressure

The pathogenesis of blue rubber bleb naevus syndrome is unclear. Although autosomal inheritance of blue rubber bleb naevus syndrome has been identified to be associated with chromosome 9p (familial), however the majority of the cases are sporadic [4]. It was found that c-kit was detected predominantly in smaller vessels within BRBNS tissues, suggesting that the stem cell factor/c-kit signaling axis may be involved in the constant growth of venous malformations [4].

Genome-wide scans permitted to identify the mutated gene in this kind of case which is TIE2/TEK, located on 9p21-22 [8]. The most common mutation causes an Arg849-to-Trp substitution (R849 W) in the intracellular kinase domain of the membrane bound tyrosine kinase receptor [8]. TIE2 signaling pathway is crucial for angiogenesis and vascular maturation. The receptor substitutions affect intracellular signaling thereby altering endothelial migration, vascular sprouting, maturation, and stability [8].

The clinical manifestations of BRBNS vary accordingly to involvement of the organs. Majority of the time cutaneous lesions are asymptomatic however some patients complain of painful lesions. Our patient complained of painful lesions over bilateral foot sole which requires sclerotherapy.

Gastrointestinal venous malformation may appear from the mouth to the anus and patient of ours has multiple naevi over small bowel, large bowel and the appendix. However small intestines has been reported to be the commonest location and lesions over the colon have left-sided predilection [5]. Typical mucosal nodule may vary from bluish tinge, bluish-red spot or polypoid [5]. The most common symptoms in the gastrointestinal (GI) tract are bleeding and secondary iron deficiency anaemia (IDA) [4], which occurred in this patient and due to life threatening condition she underwent multiple enterotomies, colostomies and appendicectomy.

The diagnosis of BRBNS can be made based on clinical judgement of cutaneous lesions with or without GIT bleeding and/or the involvement of other organs [4,5]. Gastrointestinal lesions can be examined with the help of push endoscopy, as for this patient she already underwent OGDS, colonoscopy and capsule endoscopy. Argon plasma coagulation, laser photocoagulation, sclerotherapy, mucosal resection or band ligation are frequently necessary in treating bleeding gastrointestinal venous malformations [4,5]. Imaging such as ultrasonography, CT and magnetic resonance are also beneficial in terms of diagnosing BRBNS.

Clinicians should be able to differentiate between BRBNS and hereditary hemorrhagic telangiectasia (Osler-Weber-Rendu syndrome), and Maffucci syndrome and Klippel-Trenaunay syndrome. Osler-Weber-Rendu syndrome is characterized by positive family history, bleeding punctiform angiomas, recurrent epistaxis and telangiectasia. While in Maffucci syndrome presents with bone malformations and chondrodysplasia despite having diffuse skin and soft tissue vascular malformations. Klippel-Trenaunay-Weber syndrome is characterized by hypertrophia, soft tissue and bone deformities and varicosities [4].

Treatment for gastrointestinal BRBNS is depending on the severity of the disease. The skin and soft tissue lesions rarely cause debilitating disease and are mostly a cosmetic concern [10]. In contrast, the GI lesions are a major cause of morbidity [10]. Patients usually develop severe chronic iron deficiency anemia, requiring multiple transfusions due to persistent GI losses. Minor and intermittent bleeding only requires conservative treatment such as iron supplements and blood transfusions, which occurred in the early phase of our patient’s disease progression. For serious hemorrhages, surgical resection, endoscopic sclerosis, and laser photocoagulation have been proposed and patient of ours underwent all the modalities mentioned [4]. Administration of corticosteroids and interferon has been proposed however some literatures claimed patients with BRBNS didn’t respond well to these medications and lesion regrew after the medication was stopped [4,11].

Patient of ours had sclerotherapy administration bilateral cheek lesions with mixture of sodium tetradecyl and Lipiodol. Another sclerosant agent that has been used is polidocanol and new form of polidocanol which is the foam has been reported to be superior in efficacy in comparison to fluid polidocanol [12]. According to literature, intraoral venous malformations has been treated with argon laser and the neodymium :YAG laser with promising results which penetrates up to 1 cm of soft tissue [13]. If conservative therapy is unsuccessful, resection is the option. Intervention need to be done for those lesions that cause cosmetic deformity even though it is asymptomatic lesion.

Yuksekkaya et al. reported a new method of treating bleeding venous malformation by administering Sirolimus (SRL) [10,14]. SRL is an immunosuppressant which exhibit antiangiogenic and antineoplastic properties [11]. According to an animal study, mammalian target of rapamycin (mTOR) functions as a checkpoint key in the cell cycle, acts by regulating vascular-endothelial-growth factor (VEGF) [11]. SRL acts by inhibiting the tyrosine-kinase mediated pathways and affecting the mTOR therefore downregulate the VEGF [11,14]. The knockout VEGF mice unable to develop new blood vessels when exposed to SRL [11]. Another factor that plays a role in angiogenesis is the fatty acid binding protein 4 (FABP4) which is regulated by mTOR and can be inhibited by SRL [11,14]. It functions via the stem cell factor or pathway of the c-kit, and in BRBNS there are abundance of c-kit protein which suggest this pathway is contributing to the development of the lesion.

To date, no withdrawal nor rebound symptoms reported once SRL has been discontinued. However, adverse events such as nausea, diarrhea and hypercholesterolemia has been reported. Therefore the use of SRL in BRBNS is safe provided the cholesterol level is monitored along the way [11]. Special precautions need to be taken for patients on SRL in view of predisposition to skin cancers and infections. Therefore SRL could become as an alternative treatment in managing BRBNS, however surgery is the mainstay in intractable bleeding venous malformation in GIT.

Patients with BRBNS most of the time may live along life, unfortunately their quality of life may not be optimum due to morbidity of the disease as well as the treatment itself. Therefore, prompt diagnosis, treatment and proper management of BRBNS might improve the quality of life of the patients.

## 4 Conclusion

Blue rubber bleb nevus syndrome is an atypical form of venous malformation. Misdiagnosed and suboptimal management of this disease may lead to a lot of morbidities or even mortality. Therefore, for those patients who are suspected for Blue rubber bleb nevus it is advisable to go through proper screening of upper and lower gastrointestinal scope. Thus, multidisciplinary approach and life-long follow up is recommended to ensure patient’s general well-being.

## Funding

There is no funding for this write up.

## Ethical approval

Ethical approval does not required as this is a case report.

## Consent

Patient has consented for this write up. However patient detail was not included in this write up.

## Author contribution

Fatimah Mat Johar, Salmi Mohamed Sukor, Normala Basiron Wan Azman Wan Sulaiman Arman Zaharil Mat Saad involve in managing the patient and writing the initial part of the case report as well as in the writing the discussion. Firdaus Hayati Nik Amin Sahid has contributed to modify, rearrange and the discussion of the write up.

## Registration of research studies

NA.

## Guarantor

Nik Amin Sahid.

## Provenance and peer review

Editorially reviewed, not externally peer-reviewed.

## Declaration of Competing Interest

There is no conflict of interest in this write up.
